# Protein tyrosine phosphatase PTPN3 promotes drug resistance and stem cell-like characteristics in ovarian cancer

**DOI:** 10.1038/srep36873

**Published:** 2016-11-11

**Authors:** Shuqin Li, Jian Cao, Wei Zhang, Fan Zhang, Guantai Ni, Qian Luo, Man Wang, Xiang Tao, Hongping Xia

**Affiliations:** 1 Department of Gynecology and Obstetrics, Yijishan Hospital of Wannan Medical College, Wuhu, China; 2 Department of Gynecology and Obstetrics, Nanjing Maternal and Children Care Hospital Affiliated to Nanjing Medical University, Nanjing, China; 3 Department of Pathology, Yijishan Hospital of Wannan Medical College, Wuhu, China; 4 Department of Gynecology and Obstetrics, Xiaogan Central Hospital, Xiaogan, China; 5 Department of Pathology, Obstetrics and Gynecology Hospital of Fudan University, Shanghai, China; 6 Department of Pathology, Sir Run Run Hospital & Nanjing Medical University, Nanjing, China

## Abstract

The current standard treatment for ovarian cancer is aggressive surgery followed by platinum-based combination chemotherapy. Recurrence and chemotherapeutic drug resistance are the two main factors that account for the high mortality of most ovarian cancers. Liposomal doxorubicin is primarily used for the treatment of ovarian cancer when the disease has progressed after platinum-based chemotherapy. However, relatively little is known about the genomic changes that contribute to both cisplatin and doxorubicin resistance in high-grade serous ovarian cancer (HGSC) under the selective pressure of chemotherapy. Here, we found that protein tyrosine phosphatase PTPN3 gene expression was substantially increased in both cisplatin and doxorubicin-resistant ovarian cancer cells. Silencing of PTPN3 restored sensitivity to cisplatin and doxorubicin in resistant ovarian cancer cells. Down-regulation of PTPN3 also inhibited cell cycle progression, migration, stemness *in vitro* and the tumorigenicity of resistant ovarian cancer cells *in vivo*. Meanwhile, the expression of PTPN3 was found to be regulated by miR-199 in resistant ovarian cancer cells. These findings suggest that PTPN3 promotes tumorigenicity, stemness and drug resistance in ovarian cancer, and thus is a potential therapeutic target for the treatment of ovarian cancer.

Ovarian cancer, the most common cause of gynaecologic cancer-associated death, is responsible for approximately 14,000 deaths in the United States annually^1^. Patients with advanced-stage high-grade serous ovarian cancer (HGSC) have experienced little improvement in overall survival^2^. The standard treatment is aggressive surgery followed by platinum-based combination chemotherapy. Recurrence and chemotherapeutic drug resistance are the two main factors that account for the high mortality of most ovarian cancers. After chemotherapy, platinum resistance occurs in approximately 25% of patients within six months^3^. A report on the integrated genomic analyses of ovarian carcinoma by The Cancer Genome Atlas Research Network showed that HGSC is characterised by TP53 mutations in almost all tumours (96%). BRCA1 and BRCA2 are mutated in 22% of tumours, owing to a combination of germline and somatic mutations. Low prevalence mutations include RB1, NF1, FAT3, CSMD3, GABRA6 and CDK12. The commonly dysregulated signalling pathways, such as RB, RAS/PI3K, FOXM1 and Notch, may provide promising opportunities for the treatment of advanced ovarian cancer^4^.

A recent study on the whole-genome characterisation of chemoresistant ovarian cancer by The Australian Ovarian Cancer Study Group showed that CCNE1 amplification is common in primary resistant and refractory disease. Acquired chemotherapy resistance is mainly associated with the inactivation of tumour suppressors such as RB1, NF1, RAD51B and PTEN, germline BRCA1 or BRCA2 mutations, loss of BRCA1 promoter methylation, and overexpression of the drug efflux pump MDR1^2^. Doxil, a pegylated (polyethylene glycol coated) liposome-encapsulated form of doxorubicin, is primarily used for the treatment of ovarian cancer where the disease has progressed or recurred after platinum-based chemotherapy^5^. Liposomal doxorubicin can be used together with platinum if the recurrence occurs after more than 6 months, or liposomal doxorubicin can be given alone if the recurrence occurs within 6 months of the initial or subsequent complete clinical response to platinum-containing chemotherapy^6^. However, relatively little is known of the genomic changes that contribute to both cisplatin and doxorubicin resistance of HGSC under the selective pressure of chemotherapy.

Here, we undertook a search for candidate genes that could confer both cisplatin and doxorubicin resistance in established resistant ovarian cancer cells. Using genomic analyses, we found that protein tyrosine phosphatase non-receptor type 3 (PTPN3) gene expression was substantially increased in both cisplatin and doxorubicin-resistant cells. Moreover, PTPN3 silencing restored the sensitivity of resistant ovarian cancer cells to cisplatin and doxorubicin. Silencing of PTPN3 also inhibited cell cycle progression, migration and stemness, and reduced the tumorigenicity of resistant ovarian cancer cells. These findings identify PTPN3 as a potential therapeutic target for ovarian cancer treatment and for overcoming cisplatin and doxorubicin resistance.

## Results

### PTPN3 is highly expressed in cisplatin and doxorubicin resistant ovarian cancer cells

**Figure 1:**
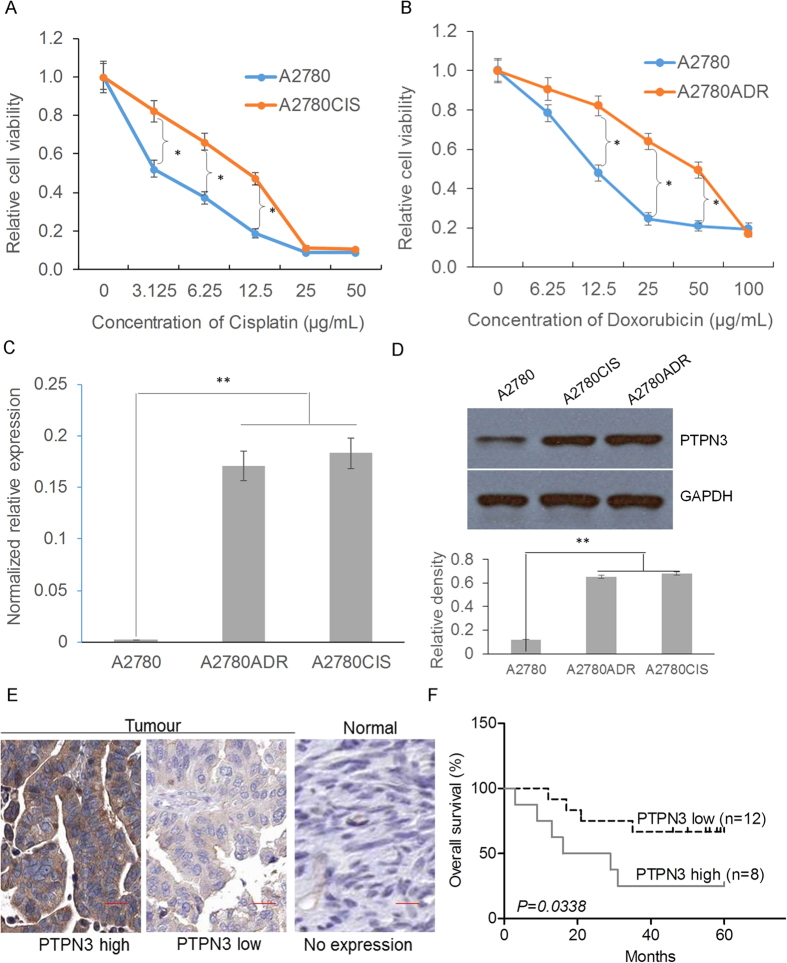
PTPN3 is highly expressed in cisplatin and doxorubicin resistant ovarian cancer cells. (**A,B**) Comparison of the sensitivity of A2780 cells to that of A2780CIS and A2780ADR cells to cisplatin (**A**) and doxorubicin (**B**). *P < 0.05 (*t* test). (**C,D**) The expression of PTPN3 in cisplatin and doxorubicin resistant ovarian cancer cells was examined by qRT-PCR (**C**) and western blotting (**D**). PTPN3 was found to be highly expressed in cisplatin and doxorubicin resistant ovarian cancer cells. **P < 0.01 (*t* test). The gels were run under the same experimental conditions. Densitometric analysis was performed to quantify the bands. (**E**) Representative immunohistochemical (IHC) images of PTPN3 in ovarian cancer tissue samples. Scale bar = 50 μm. (**F**) The high expression of PTPN3 was significantly associated with poor overall survival of ovarian cancer patients.

To identify the critical factors associated with both cisplatin and doxorubicin resistance in ovarian cancer, we compared the gene expression differences in A2780, A2780CIS and A2780ADR cells obtained from the European Collection of Cell Cultures (ECACC) using ovarian cancer cell line panel gene expression data^7^. Among the panel of significantly different genes, PTPN3 was significantly overexpressed in both A2780CIS and A2780ADR cells compared to A2780 cells (Table S1). The resistance of A2780CIS and A2780ADR to cisplatin and doxorubicin was confirmed by the MTS assay (Fig. 1A,B). The significantly increased expression of PTPN3 in both A2780CIS and A2780ADR was further validated by real-time quantitative reverse transcription PCR (qRT-PCR) and western blotting (Fig. 1C,D). Importantly, the highly increased expression of PTPN3 was also detected in approximately one third of the clinical ovarian cancer tissue samples, but not detectable in normal ovarian tissue samples (Fig. 1E). The high expression of PTPN3 was significantly associated with poor overall survival of ovarian cancer patients (Fig. 1F). These data suggest that overexpression of PTPN3 may play a critical role in mediating both cisplatin and doxorubicin resistance in ovarian cancer cells.

### Silencing of PTPN3 inhibits cell cycle progression in resistant ovarian cancer cells

**Figure 2:**
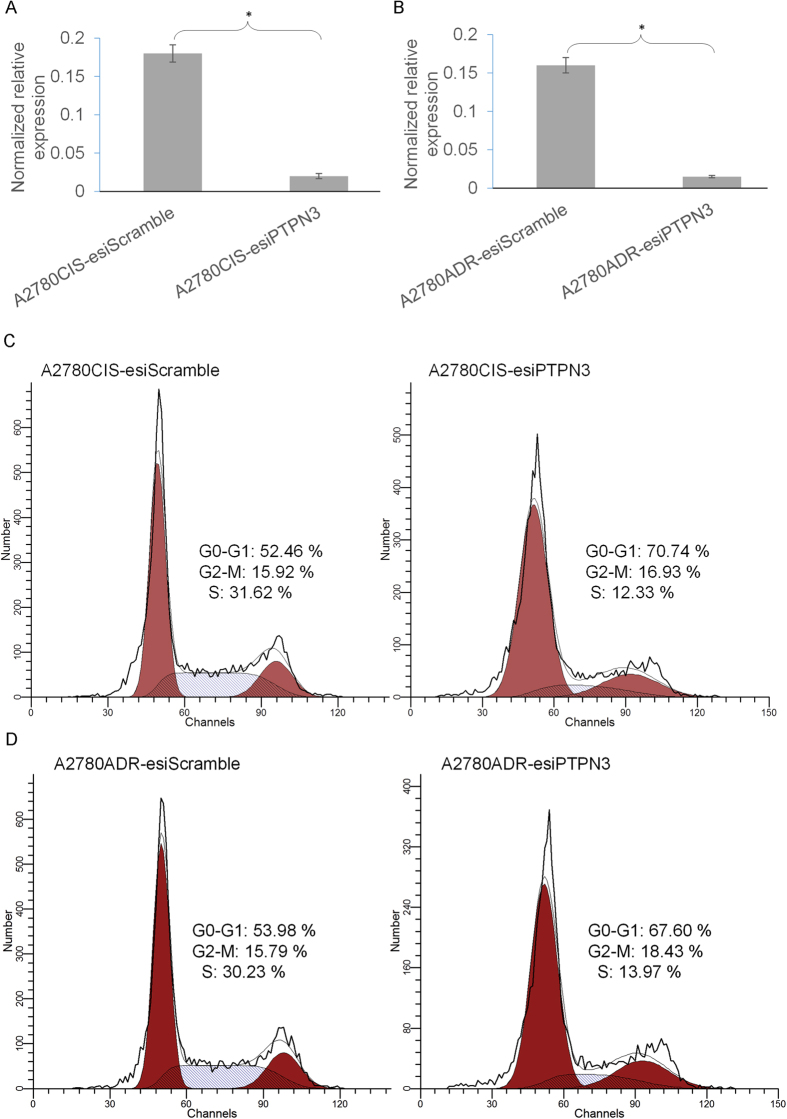
Silencing of PTPN3 inhibits cell cycle progression in resistant ovarian cancer cells. (**A,B**) The expression of PTPN3 was significantly silenced by PTPN3 esiRNA transfection using qRT-PCR analysis in both A2780CIS (**A**) and A2780ADR (**B**) cells. *P < 0.05 (*t* test). (**C,D**) Representative cell cycle analysis by flow cytometry shows that silencing PTPN3 significantly increased the G0-G1 phase cell population and decreased the S phase cell population in both A2780CIS (**C**) and A2780ADR (**D**) cells.

Cell cycle progression has been shown to play important roles in drug resistance. To investigate the molecular mechanism of PTPN3-mediated cisplatin and doxorubicin resistance in ovarian cancer cells, cell cycle analysis was used to examine the effect of silencing PTPN3 on cell signalling pathways. After transfection with PTPN3 esiRNAs (esiPTPN3), an endoribonuclease-prepared siRNA pool comprised of a heterogeneous mixture of siRNAs that all target the same mRNA sequence of PTPN3, a significant silencing effect of PTPN3 in both cisplatin and doxorubicin resistant ovarian cancer cells was confirmed by qRT-PCR (Fig. 2A,B). Subsequent cell cycle analysis by flow cytometry indicated that silencing of PTPN3 significantly increased the G0-G1 phase cell population and decreased the S phase cell population in both cisplatin and doxorubicin resistant ovarian cancer cells. These data suggest that silencing PTPN3 inhibits cell cycle progression in resistant ovarian cancer cells.

### Silencing of PTPN3 inhibits resistant ovarian cancer cell growth, migration and drug resistance

**Figure 3:**
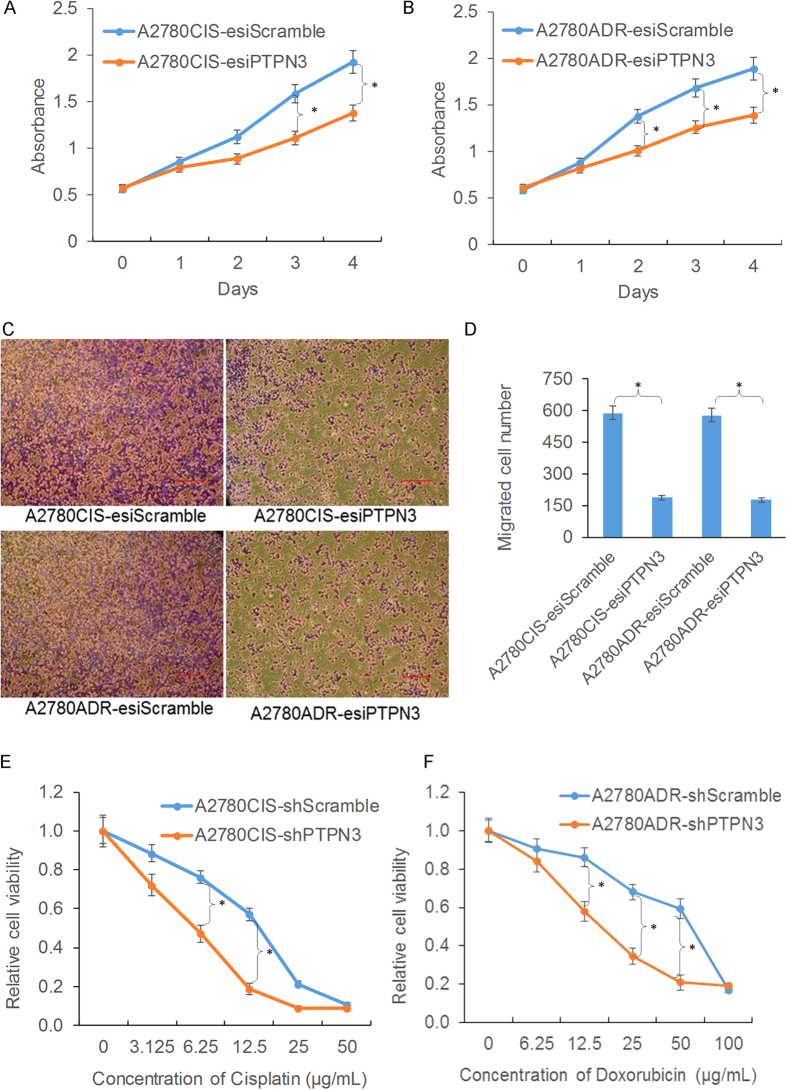
Silencing of PTPN3 inhibits resistant ovarian cancer cell growth, migration and drug resistance. (**A,B**) MTS assay results showing that cell growth was dramatically reduced by silencing PTPN3 in both A2780CIS (**A**) and A2780ADR (**B**) cells. (**C,D**) The Transwell migration assay showed that silencing PTPN3 significantly suppressed the migration rates of both cisplatin and doxorubicin resistant ovarian cancer cells. Scale bar = 100 μm. (**E,F**) MTS assay results showing that silencing PTPN3 significantly contributed to increasing the sensitivity of resistant ovarian cancer cells to cisplatin (**E**) and doxorubicin (**F**). *P < 0.05 (*t* test).

To investigate the critical role of PTPN3 in ovarian cancer drug resistance and cell cycle progression, we examined the effects of silencing of PTPN3 on resistant ovarian cancer cell growth, migration and drug resistance. The cell growth curves indicated that cell growth was dramatically reduced by silencing PTPN3 in both cisplatin and doxorubicin resistant ovarian cancer cells (Fig. 3A,B). We next examined whether PTPN3 is a critical molecule for resistant ovarian cancer cell migration using the Transwell migration assay. Silencing PTPN3 significantly suppressed the migration rates of both cisplatin and doxorubicin resistant ovarian cancer cells (Fig. 3C,D). We then assayed drug sensitivity to cisplatin and doxorubicin. The results showed that silencing PTPN3 significantly contributed to increasing the sensitivity of resistant ovarian cancer cells to cisplatin and doxorubicin (Fig. 3E,F). These data collectively indicate that silencing of PTPN3 inhibits resistant ovarian cancer cell growth, migration and drug resistance.

### Stable silencing of PTPN3 inhibits colony formation and stemness in resistant ovarian cancer cells

**Figure 4:**
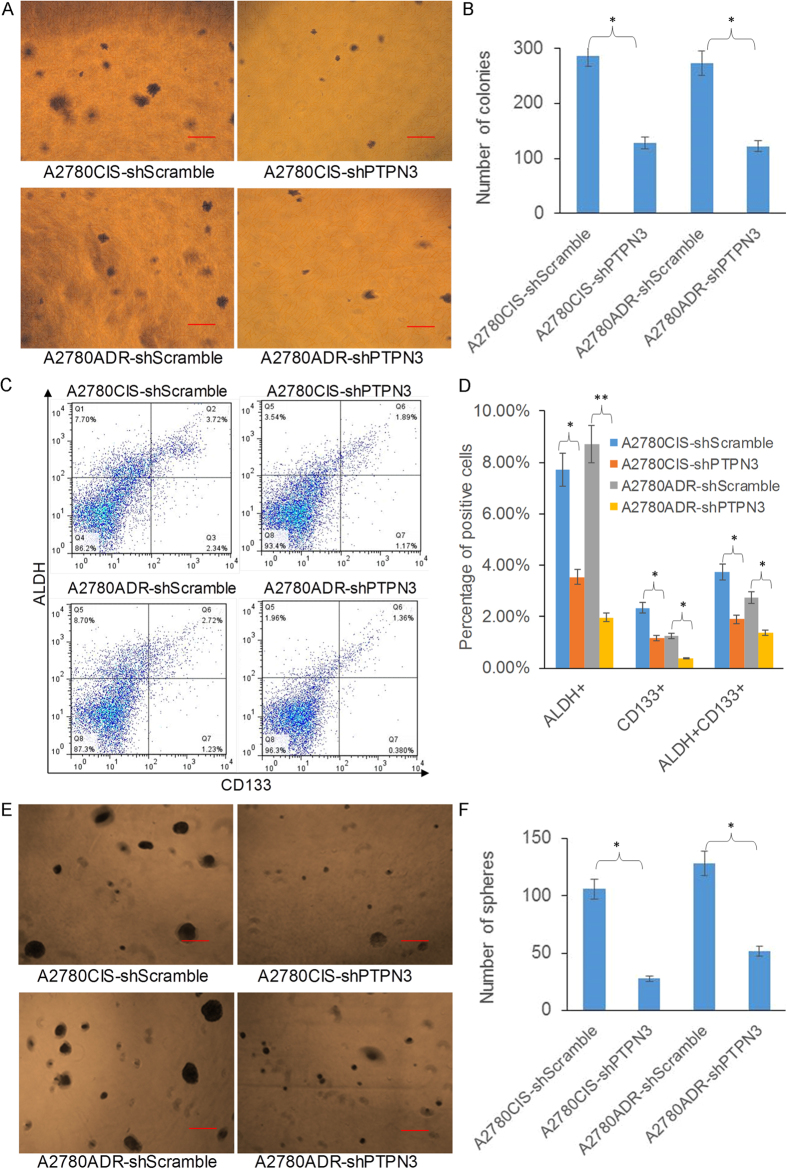
Stable silencing of PTPN3 inhibits colony formation and stemness in resistant ovarian cancer cells. (**A,B**) Representative images of the soft agar assay and the quantification of colonies show that stable silencing of PTPN3 inhibited cellular anchorage-independent growth of resistant ovarian cancer cells *in vitro*. Scale bar = 50 μm. (**C,D**) Representative flow cytometry plots and the quantification of positive cells show that stable silencing of PTPN3 significantly decreased the ALDH+, CD133+ and ALDH+ CD133+ cell populations. (**E,F**) Representative images of tumour sphere formation and the quantification of spheres showed that stable silencing of PTPN3 significantly inhibited the sphere formation ability of both cisplatin and doxorubicin resistant ovarian cancer cells. Scale bar = 50 μm. *P < 0.05 (*t* test).

Colony formation in soft agar is the most widely used assay to evaluate cellular anchorage-independent growth *in vitro*. Soft agar colony formation assays showed that stable silencing of PTPN3 significantly inhibited the colony forming ability of both cisplatin and doxorubicin resistant ovarian cancer cells, suggesting that stable silencing of PTPN3 inhibits cellular anchorage-independent growth of resistant ovarian cancer cells *in vitro* (Fig. 4A,B). Increasing evidence has suggested that the stemness of cancer cells is thought to be responsible for cancer initiation and drug resistance. Previous studies have shown that ALDH+ and CD133+ cells are enriched with ovarian cancer-initiating (stem) cells, and that ALDH and CD133 may be widely used as reliable markers to investigate ovarian cancer stem cell biology^8^. Flow cytometry analysis showed that stable silencing of PTPN3 significantly decreased the ALDH+, CD133+ and ALDH+ CD133+ cell populations (Fig. 4C,D). The tumour sphere formation assay showed that stable silencing PTPN3 significantly inhibited the sphere forming ability of both cisplatin and doxorubicin resistant ovarian cancer cells (Fig. 4E,F). These data suggest that stable silencing of PTPN3 inhibits colony formation and stemness in resistant ovarian cancer cells.

### The expression of PTPN3 is regulated by miR-199 in resistant ovarian cancer cells

**Figure 5:**
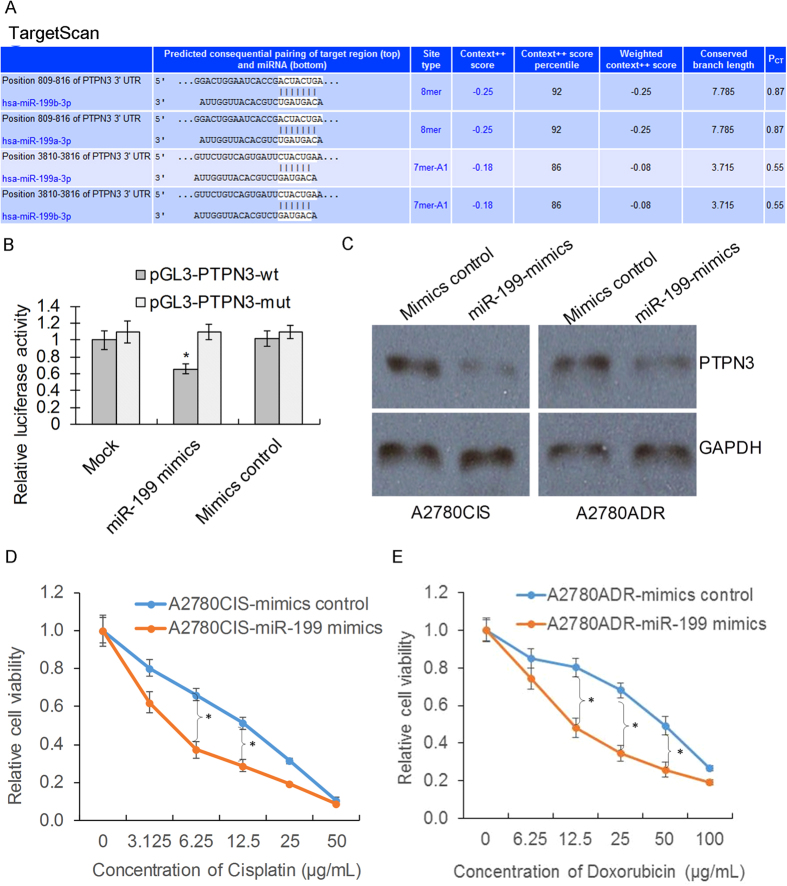
The expression of PTPN3 is regulated by miR-199 in resistant ovarian cancer cells. (**A**) The predicted binding sites of PTPN3 3′UTR and miR-199 by TargetScan. (**B**) Luciferase activity in cells co-transfected with miR-199 mimics and the pGL3-PTPN3-wt vector was significantly decreased when compared with the control. (**C**) Western blotting images showing that overexpression of miR-199 decreased the expression of PTPN3 in resistant ovarian cancer cells. The gels were run under the same experimental conditions. (**D,E**) The MTS assay showed that overexpression of miR-199 increased the sensitivity of resistant ovarian cancer cells to cisplatin (**D**) and doxorubicin (**E**). *P < 0.05 (*t* test).

Previous studies have shown that at least one-third of human genes are regulated by miRNAs^9^. After demonstrating the important roles of PTPN3 in resistant ovarian cancer cells, we next investigated whether miRNAs regulate the expression of PTPN3. To identify the potential posttranscriptional regulation of PTPN3 by miRNAs, we used two online software resources, i.e. TargetScan and miRDB, for prediction. A panel of miRNAs was predicted to be potential regulators of PTPN3 by both miRNA target prediction programs (Table S3). We then used the luciferase reporter assay to validate that PTPN3 could be potentially regulated by miR-199. To validate whether miR-199 directly recognises the 3′-UTR of PTPN3 mRNA, we cloned a sequence containing the predicted target site and a mutated sequence with the predicted target sites downstream of the pGL3 luciferase reporter gene to generate pGL3-PTPN3-wt or pGL3-PTPN3-mut vectors (Fig. 5A). The vectors were then co-transfected with the miR-199 mimics or control into HEK293 cells. A Renilla luciferase vector (pRL-TK) was used to normalise the differences in transfection efficiency. Luciferase activity in cells co-transfected with the miR-199 mimics and the pGL3-PTPN3-wt vector was significantly decreased when compared with the control (Fig. 5B). Next, we further detected the protein expression of PTPN3 in resistant ovarian cancer cells after transfection with miR-199 mimics or control. The results show that overexpression of miR-199 decreased the expression of PTPN3 in resistant ovarian cancer cells (Fig. 5C). Consistently, overexpression of miR-199 also increased the sensitivity of resistant ovarian cancer cells (Fig. 5D,E). These data suggest that the expression of PTPN3 is regulated by miR-199 in resistant ovarian cancer cells.

### Stable silencing of PTPN3 inhibits resistant ovarian cancer cell tumorigenicity *in vivo*

**Figure 6:**
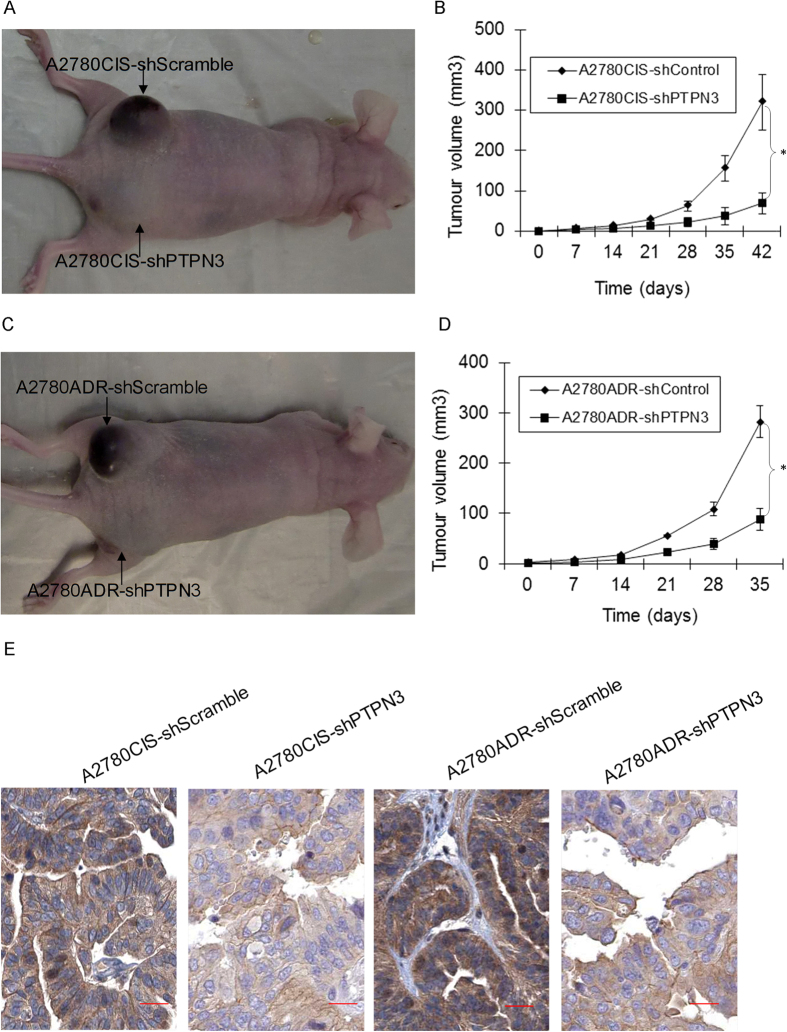
Stable silencing PTPN3 inhibits resistant ovarian cancer cell tumorigenicity *in vivo*. (**A,B**) Representative tumour images and tumour growth curved showing that the tumours formed from shPTPN3 stably transfected A2780CIS cells grew significantly more slowly than those formed from shScramble control transfected A2780CIS cells. *P < 0.05 (*t* test). (**C,D**) Representative tumour images and tumour growth curves showing that the tumours formed from shPTPN3 stable transfected A2780ADR cells grew significantly more slowly than those formed from shScramble control transfected A2780ADR cells. *P < 0.05 (*t* test). (**E**) Representative images of IHC staining of tumour tissues showing that the expression of PTPN3 was significantly decreased in shPTPN3 stably transfected tumours. Scale bar = 50 μm.

The effect of stable silencing of PTPN3 on the tumorigenicity of resistant ovarian cancer cells was further investigated in a mouse model *in vivo*. Immunodeficient Balb/C mice were subcutaneously injected with resistant ovarian cancer cells that had been previously stably transfected with PTPN3 shRNA or shScramble control. Throughout the tumorigenic period, the tumours that formed from shPTPN3 stably transfected cells grew significantly slower than those formed from shScramble control transfected cells, using both A2780CIS (Fig. 6A,B) and A2780ADR (Fig. 6C,D) cells. After 35 days, immunohistochemical (IHC) staining of tumour tissues showed that the expression of PTPN3 was significantly decreased in shPTPN3 stably transfected tumours (Fig. 6E). These data suggest that stable silencing of PTPN3 inhibits resistant ovarian cancer cell tumorigenicity *in vivo*.

## Discussion

The results reported here provide evidence that PTPN3 regulates sensitivity to cisplatin and doxorubicin in ovarian cancer cells. PTPN3 is a member of the protein tyrosine phosphatase (PTP) family, which is known to comprise signalling molecules that regulate a variety of cellular processes including cell growth, differentiation, mitosis and oncogenic transformation^10^. Here, the expression of PTPN3 was observed to be substantially increased in both cisplatin and doxorubicin resistant ovarian cancer cells through the genomic analysis of cisplatin and doxorubicin resistant cells compared with the parental ovarian cancer cells. Silencing of PTPN3 restored sensitivity to cisplatin and doxorubicin in resistant ovarian cancer cells. These data suggest that PTPN3 may play a critical role in the development of ovarian cancer and contribute to chemotherapy resistance.

Platinum-based chemotherapy is currently the first-line treatment for advanced stage HGSC patients. However, resistance to platinum-based chemotherapy is the main obstacle in clinical practice^11^. Platinum resistant ovarian cancer is comprised of a heterogeneous and complex spectrum of diseases^12^. The mechanism of platinum therapy resistance has been reported, including changes in cisplatin transport and trafficking, disruption of apoptosis, increased tolerance to cisplatin-DNA adducts and increased DNA repair in response to cisplatin-DNA interactions^13^. Many ovarian tumours exhibit multiple resistance pathways simultaneously^14^. Patients with recurrent ovarian cancer are typically categorised as having either platinum resistant or platinum sensitive disease, based on a platinum-free interval of less than or greater than 6 months, respectively^12^. A range of second-line chemotherapeutic agents has been approved for the treatment of ovarian cancer, including doxorubicin, etoposide, topotecan, gemcitabine and trabectedin^11^. Liposomal doxorubicin can be used together with platinum if the recurrence occurs after more than 6 months, or liposomal doxorubicin can be given alone if the recurrence occurs within 6 months of the initial or subsequent complete clinical response to platinum-containing chemotherapy. These are mostly used as second-line drugs, but they have been poorly effective in trials involving large unselected populations of relapsed patients^11^. Therefore, it is interesting to identify the factors contributing to both cisplatin and doxorubicin resistance in HGSC under the selective pressure of chemotherapy.

Here, we found that overexpression of PTPN3 promotes both cisplatin and doxorubicin resistance. Previous studies on the mutational analysis of the tyrosine phosphatase gene superfamily in human cancers identified 83 somatic mutations in six PTPs (PTPRF, PTPRG, PTPRT, PTPN3, PTPN13, PTPN14), affecting 26% of colorectal cancers and a smaller fraction of lung, breast and gastric cancers^15^. Recently, activating mutations in PTPN3 have been shown to promote cholangiocarcinoma cell proliferation and migration and were associated with tumour recurrence in patients. Mutations in PTPN3 have been detected in 41.1% of cholangiocarcinomas. Transgenic expression of PTPN3 in cell lines increases cell proliferation, colony formation and migration^16^. However, the mutation rate of PTPN3 is relatively low in ovarian cancer, according to the TCGA dataset. Previous studies have suggested that at least one-third of human genes are estimated to be miRNA targets^9^. We found that the expression of PTPN3 was regulated by miR-199 in resistant ovarian cancer cells. Therefore, the miR-199/PTPN3 network likely contributes to both cisplatin and doxorubicin resistance of HGSC under the selective pressure of chemotherapy.

Protein tyrosine phosphatases (PTPs) have been found to function as tumour suppressors or oncogenes, depending on the substrate involved and the cellular context. PTPN3 has been reported to be an oncogene in gastric^16^, colon^17^ and breast cancer^18^. However, a recent study showed that PTPN3 inhibits lung cancer cell proliferation and migration by promoting the endocytic degradation of EGFR, indicating that PTPN3 may act as a tumour suppressor in lung cancer through its modulation of EGFR signalling^19^. In the present study, highly increased expression of PTPN3 was also detected in around one third of clinical ovarian cancer tissue samples, but was not detectable in normal ovarian tissue samples. Moreover, high expression of PTPN3 was significantly associated with poor overall survival in ovarian cancer patients. Silencing PTPN3 inhibits resistant ovarian cancer cell growth, cell cycle progression, migration, drug resistance, stemness and tumorigenicity. These data suggest that PTPN3 is an oncogene that promotes drug resistance and stem cell-like characteristics in ovarian cancer, and thus it is a potential therapeutic target for the treatment of ovarian cancer.

## Materials and Methods

### Cell lines and cell culture

The human epithelial serous ovarian cancer cell lines A2780 (cisplatin-sensitive) was purchased from the European Collection of Cell Cultures (ECACC, UK) and cultured in a humidified atmosphere at 37 °C, 5% CO_2_. They were maintained in RPMI 1640 medium supplemented with 10% foetal bovine serum (FBS) and 1% penicillin/streptomycin mixture (Thermo Fisher Scientific, USA). Cisplatin and doxorubicin were obtained from Sigma-Aldrich and dissolved in DMSO. Cisplatin and doxorubicin resistant cells were developed from their parental cell line A2780 by gradual incremental doses of cisplatin or doxorubicin administration in cell culture medium in subsequent passages for about 6 months. Thereafter cisplatin resistant A2780CIS and doxorubicin resistant A2780ADR cells were maintained in presence of 1 μg/ml cisplatin or doxorubicin in cultured medium for 72 h in every alternate passage in accordance with the ECACC guidelines.

### Animal studies

The validated shRNA stable transfected A2780CIS and A2780ADR cells were re-suspended in PBS and implanted into the right and left flanks (5 × 10^6^ cells per flank) of female BALB/c nude mice via subcutaneous injections. The study was approved by the Animal Ethics Committee of Yijishan Hospital Affiliated to Wannan Medical College, China. All animal related methods were performed in accordance with the relevant guidelines and regulations. Tumor volumes were determined each week by measuring their length (a) and width (b) using a vernier caliper. The tumor volume (V) was calculated according to the formula V = ab^2^/2. The statistical significance between tumor sizes in the shPTPN3 and shScramble control transfected groups was evaluated using the Student’s t test. The tumors were fixed in 10% neutral buffered formalin before being processed into paraffin blocks. The tissue sections were stained with PTPN3 primary antibody (Abcam) using standard IHC techniques.

### Immunohistochemistry (IHC)

The tissue samples were collected and all experimental protocols were approved by the Committees for Ethical Review of Research Involving Human Subjects of Yijishan Hospital Affiliated to Wannan Medical College. Informed consent was obtained from all subjects. All experiments were performed in accordance with relevant guidelines and regulations. Paraffin embedded tissue samples from consented patients or xenograft mouse tissues derived from A2780CIS and A2780ADR transfected cells were cut into 5μm sections and placed on poly-lysine-coated slides. The slides were then deparaffinised in xylene and rehydrated using a series of graded alcohol. Antigen retrieval was performed by heat mediation in citrate buffer (pH 6; Dako). Samples were blocked with 10% goat serum before incubation with primary antibody. The samples were incubated overnight using following primary antibodies: rabbit anti-PTPN3 (1:100) or an isotype-matched IgG (Sigma) as a negative control in a humidified container at 4 °C. Immunohistochemical staining was performed with the Dako Envision plus System (Dako, Carpinteria, CA, USA) according to the manufacturer’s instructions. The intensity of staining was evaluated by digital image analysis on the scale of 0 to 4 according to the percentage of positive tumors (Low expression: 0, negative control; 1, 0–10%; 2, 10–25%; High expression: 3, 25–50% and 4, >50%).

The rest of materials and methods are provided in the online supplementary information.

## Additional Information

**How to cite this article**: Li, S. *et al*. Protein tyrosine phosphatase PTPN3 promotes drug resistance and stem cell-like characteristics in ovarian cancer. *Sci. Rep*. **6**, 36873; doi: 10.1038/srep36873 (2016).

**Publisher’s note:** Springer Nature remains neutral with regard to jurisdictional claims in published maps and institutional affiliations.

## Supplementary Material

Supplementary Information
