# Supplementary material for: Protein tyrosine phosphatase PTPN3 promotes drug resistance and stem cell-like characteristics in ovarian cancer
Source: Sci Rep. 2016 Nov 11;6:36873. doi: 10.1038/srep36873 (PMC5105059; doi:10.1038/srep36873)
Supplement: Supplementary Information [file srep36873-s1.pdf]

Supplementary information

Protein tyrosine phosphatase PTPN3 promotes drug resistance and stem cell-like characteristics in ovarian cancer

Shuqin Li[1,*], Jian Cao[2,*], Wei Zhang[3], Fan Zhang[3], Guantai Ni[1], Qian Luo[1], Man Wang[4], Xiang Tao [5,#], Hongping Xia[3,6,#]

Supplementary materials and methods

CellTiter 96 AQueous One solution cell proliferation assay

After treatment, the cell growth and viability to drug treatment was measured using the CellTiter 96 AQueous One solution cell proliferation assay (Promega, USA). This assay is based on the cleavage of tetrazoliumsalts [3-(4,5-dimethyl-2-yl)-5-(3-carboxymethoxyphenyl)-2-(4-sulfophenyl)-2H-tetrazolium, inner salt; MTS] and an electron coupling reagent (phenazineethosulfate; PES). The absorbance was read at 490 nm using a Wallac VICTOR3. multilabel plate reader from PerkinElmer followed by 2 hours incubation with CellTiter 96 AQueous One solution in culture medium. Results were normalized against controls with illumination and presented as percentage of cell viability.

Real time quantitative reverse transcription PCR (qRT-PCR) analysis

The total RNA was isolated using the RNeasy Mini Kit (QIAGEN) and examined using the NanoDrop ND-1000 UV-Vis Spectrophotometer. For mRNA detection, the total RNA was reverse transcribed using the 5X All-In-One RT MasterMix (Applied Biological Materials Inc. Canada). The qPCR was performed using EvaGreen 2X qPCR MasterMix (Applied Biological Materials Inc. Canada). For miRNA detection, the total RNA samples were polyadenylated and reverse transcribed for a two-step quantitative RT-PCR reaction using the miRNA cDNA Synthesis Kit and miRNA qPCR MasterMixes (Applied Biological Materials Inc. Canada) according to the manufacturer's instructions. The *HPRT1* or *U6* gene was used as an endogenous control, and fold changes were calculated via relative quantification ($2^{-\Delta Ct}$).

Western blotting

The cells were plated in the six well plate and were allowed to grow to 90% confluent within 3 days. Cell lysate was prepared in RIPA buffer with the addition of protease inhibitor cocktail, phosphatase inhibitor cocktail and DTT (Dithiothreitol) (Sigma Aldrich, St. Louis, MO, USA). Protein concentration was calculated by the BCA Protein assay kit (Thermo Scientific, UK). NuPAGE Novex 4-12% Bis-Tris protein gels (Novex, Life Technologies) were used for all Western blotting assays. The PTPN3 and GAPDH primary and secondary antibody were bought from Sigma. Bands were detected using super signal west pico chemiluminescent substrate (Thermo Scientific, USA) after incubation with primary antibody and HRP-cojugated secondary antibody (Sigma).

Cell cycle analysis

The A2780CIS and A2780ADR cells were seeded in the six well plates and transfected with esiRNA for 72 h. Cells were collected by tripsinization and washed twice in PBS, fixed in 70% chilled ethanol by adding dropwise into the samples while vortexing and stored at 4 °C until analyzed. Immediately before analysis, cells were washed twice in PBS, and incubated with 250 μg/ml RNase A (Invitrogen) for 30 min at room temperature, followed by staining with PI (Sigma) at 20 μg/ml final concentration for 30 min incubation in dark. The cell cycle distribution and percentage of apoptotic cells were analysed using a FACS analyser (BD LSRFortessa). Ten thousand cells were analysed for each sample. Appropriate gating was used to select the single-cell population. The percentage of cells in sub-2n phase was determined using software (BD FACSDiva v6.0).

In vitro transwell cell migration assay

The transwell cell migration assay was carried out using 24 well Transwell chambers (8 μm pore size, BD Biosciences, CA, USA). In brief, 600 μl complete medium was added to the bottom chamber. The A2780CIS and A2780ADR cells were seeded in the six well plates and transfected with esiRNA for 24 h. The transfected cells were suspended in serum-free medium, and 500 μl of the cell suspension (containing 5×10^4 cells) was placed in the upper chamber. After 24 hours, any non-migrated cells on the upper surface of the membrane were removed using a cotton swab, and the cells on the bottom surface of the membrane were fixed in 95% ethanol and stained with a 0.1% crystal violet solution. The stained migrated cells adhering to the

bottom surface of the membrane were photographed and counted in five randomly selected areas under a 40 × microscope field. Each experiment was repeated three times.

Soft agar colony formation assay

The A2780CIS and A2780ADR cells was seeding in the six well plates and transfected with validated shRNA shPTPN3 or shScramble (Sigma). The stable transfected cell clones were selected using puromycin (2ug/ml). The validated shRNA stable transfected A2780CIS and A2780ADR cells were seeded in six well plates in growth medium containing 0.7% soft agar (1ml per well) on top of a layer of growth medium containing 1.4% agar (1ml per well). Growth medium (1ml) with 10% FBS was added on top of the agar. The cell suspension was plated and cultured in a 37 ^{o}C incubator for around 3 weeks. After that, the colonies were fixed with methanol and stained with 0.05% crystal violet in 25% methanol. The colonies were pictured and counted under an inverted microscope.

Flow cytometric analysis of ALDH+ and CD133+ cell population

The validated shRNA stable transfected A2780CIS and A2780ADR cell suspensions were counted and incubated with primary antibodies CD133 (Miltenyi Biotec). ALDH+ enzymatic activity was defined using the ALDEFLUOR kit per protocol (Stem Cell Technologies, Vancouver, BC, Canada). For each sample ½ of cell/substrate mixture was treated with 50 mmol/L diethylaminobenzaldehyde (DEAB). Cells were incubated for 45 min. Gating was established using Propidium Iodide (PI)-exclusion for viability and ALDEFLUOR/DEAB

treated cells were used to define negative gates. FACS was performed with ≥1×105 cells using the BD FACSCanto II (Becton Dickinson, San Diego, USA)[1].

Sphere formation

Sphere culture was performed as previously described[2]. Briefly, the validated shRNA stable transfected A2780CIS and A2780ADR cells were plated in triplicate in ultra-low attachment plates in serum-free DMEM/F12 medium supplemented supplemented with 5 μg/mL insulin (Sigma), 20 ng/mL human recombinant epidermal growth factor (EGF, Peprotech), 10 ng/mL basic fibroblastic growth factor (bFGF, Peprotech) and B27 Supplement (Gibco). Cells were plated at the indicated density and from 1,000–10,000 cells/ml in subsequent passage. Sphere formation was assessed 2 weeks after seeding the cells.

Luciferase reporter assay

The 3'-UTR sequence of PTPN3 predicted to interact with miR-199 or a mutated 3'-UTR sequence within the predicted target sites was synthesized and inserted into the XbaI and FseI sites of the pGL3 control vector (Promega, Madison, WI). These constructs were named as pGL3-PTPN3-3'UTR or pGL3-PTPN3-3'UTR-mut, respectively. For the reporter assay, HEK293 cells were plated onto 24-well plates and transfected with the above constructs and miR-199 mimics or mimics control using Lipofectamine 3000 (Thermo Fisher Scientific, USA). A Renilla luciferase vector pRL-SV50 (Promega, Madison, WI) was co-transfected to normalize the difference in the transfection efficiency. After 48h, the cells were harvested and assayed using the dual-luciferase reporter assay system (Promega, Madison, WI) according to the

manufacturer's instructions. Results were obtained from three independent experiments performed in duplicate.

Survival and statistical analysis

The experimental data are presented as the mean ± standard deviation (SD). All statistical analyses were performed using ANOVA or a two-tailed Student's *t* test by GraphPad Prism 5.0 (GraphPad Software, La Jolla, CA). The survival curves were created using the Kaplan-Meier method and statistically compared using a log-rank test. Differences were considered statistically significant when the P-values were less than 0.05.

References:

1 Silva, I. A. *et al.* Aldehyde dehydrogenase in combination with CD133 defines angiogenic ovarian cancer stem cells that portend poor patient survival. *Cancer research* **71**, 3991-4001 (2011).

2 Xia, H. *et al.* Loss of brain-enriched miR-124 microRNA enhances stem-like traits and invasiveness of glioma cells. *J Biol Chem.* **287**, 9962-9971 (2012).

Supplementary Tables

Table S1 The significantly dysregulated genes of A2780CIS and A2780ADR compared with A2780 in the GSE53418

Probeset ID	Gene Symbol	A2780_ADR vs. A2780	A2780_CIS vs. A2780
3219944	PTPN3	100.481	101.965
2965223	EPHA7	-106.497	-100.621
3344161	NAALAD2	-115.539	-103.283
3986112	NRK	-113.747	-108.412
2965237	EPHA7	-107.005	-108.622
3510132	TRPC4	-171.893	-109.148
2366851	PRRX1	-132.973	-115.105
2965214	EPHA7	-129.582	-115.157
3510131	TRPC4	-121.894	-115.816
3324451	FIBIN	-136.241	-116.184
3652015	OTOA	-111.461	-116.226
3366944	SLC5A12	-112.916	-116.37
3986115	NRK	-209.599	-117.304
3324365		-113.59	-117.724
3510139	TRPC4	-145.005	-121.646
3777322	ARHGAP28	-148.227	-126.402
3986123	NRK	-126.314	-129.584
3777312	ARHGAP28	-134.569	-131.624
2342505	LHX8	-114.281	-139.226
3986143	NRK	-202.703	-139.253
3986026	IL1RAPL2	-149.324	-139.482
3510167	TRPC4	-105.298	-140.921
3486124	FREM2	-120.302	-145.653
3344158	NAALAD2	-239.081	-145.898
2342510	LHX8	-134.037	-147.594
2965210	EPHA7	-284.958	-150.006
3986136	NRK	-106.805	-150.631
3344160	NAALAD2	-162.891	-152.248
3986111	NRK	-303.754	-152.345
3986122	NRK	-116.957	-155.158
3652036	OTOA	-153.474	-158.502
3986109	NRK	-123.26	-159.051
3324346	ANO3	-257.755	-160.76

3510168	TRPC4	-157.404	-171.234
3344156	NAALAD2	-122.034	-171.54
3103712	PI15	-190.764	-175.245
3324344	ANO3	-113.016	-180.912
3344155	NAALAD2	-289.291	-193.526
3344179	NAALAD2	-167.326	-203.439
3777317	ARHGAP28	-160.854	-225.81
3986117	NRK	-204.776	-227.574
3324387	ANO3	-265.113	-235.006
3324390	ANO3	-220.355	-237.983
2342509	LHX8	-186.281	-246.597
3777311	ARHGAP28	-174.349	-253.825
2372798	RGS1	-101.795	-269.696
3103714	PI15	-108.553	-281.903
3986108	NRK	-188.003	-292.674
3424902	NTS	-141.583	-313.984
3777313	ARHGAP28	-149.726	-319.436
2965209	EPHA7	-141.259	-323.718
3986121	NRK	-121.908	-326.175
3103706	PI15	-192.921	-338.613
3324362	ANO3	-316.979	-343.539
3024060	MEST	-209.537	-357.971
3103717	PI15	-126.006	-372.912
3986116	NRK	-386.658	-406.543
3324450	FIBIN	-513.827	-418.102
3986141	NRK	-253.374	-458.852
3103713	PI15	-169.743	-479.241
2372794	RGS1	-659.785	-537.644

Table S2 PTPN3 Expression and Clinic Pathological Characteristics

**Variable**	**PTPN3**		
	**Low**	**High**	***P* value**
Age			
≤58	5	3	
>58	7	5	0.282
FIGO stage			
Early (I/II)	8	2	
Late (III/IV)	4	6	0.026
Histology			
Serous	7	5	
Mucinous	5	3	0.162
Grade			
I/II	7	3	
III/IV	5	5	0.038

Note: FIGO: The International Federation of Gynecology and Obstetrics. Analysis with X^2 test.

Table S3 Predicted microRNA targeting PTPN3-TargetScan and miRDB

**miRNA**	**Position in the UTR**	**Seed match**	**Target Score in miRDB**
hsa-miR-106a-5p	128-135	8mer	97
hsa-miR-106b-5p	128-135	8mer	97
hsa-miR-129-1-3p	416-422	7mer-m8	52
hsa-miR-129-2-3p	416-422	7mer-m8	52
hsa-miR-135a-5p	1429-1435	7mer-m8	69
hsa-miR-135b-5p	1429-1435	7mer-m8	69
hsa-miR-153-3p	3849-3856	8mer	85
hsa-miR-15a-5p	832-839	8mer	98
hsa-miR-15b-5p	832-839	8mer	98
hsa-miR-16-5p	832-839	8mer	98
hsa-miR-17-5p	128-135	8mer	97

hsa-miR-195-5p	832-839	8mer	98
**hsa-miR-199a-3p**	**3810-3816**	**7mer-1A**	**82**
**hsa-miR-199b-3p**	**3810-3816**	**7mer-1A**	**82**
hsa-miR-20a-5p	128-135	8mer	97
hsa-miR-20b-5p	128-135	8mer	99
hsa-miR-3129-5p	3810-3816	7mer-1A	82
hsa-miR-424-5p	832-839	8mer	100
hsa-miR-497-5p	832-839	8mer	98
hsa-miR-519d-3p	128-135	8mer	97
hsa-miR-526b-3p	128-135	8mer	97
hsa-miR-543	3774-3780	7mer-m8	83
hsa-miR-6838-5p	832-839	8mer	98
hsa-miR-93-5p	128-135	8mer	97
